# Online Multi-Modal Robust Non-Negative Dictionary Learning for Visual Tracking

**DOI:** 10.1371/journal.pone.0124685

**Published:** 2015-05-11

**Authors:** Xiang Zhang, Naiyang Guan, Dacheng Tao, Xiaogang Qiu, Zhigang Luo

**Affiliations:** 1 Science and Technology on Parallel and Distributed Processing Laboratory, College of Computer, National University of Defense Technology, Changsha, Hunan, China; 2 The Centre for Quantum Computation & Intelligent Systems and the Faculty of Engineering and Information Technology, University of Technology, Sydney, 81 Broadway Street, Ultimo, NSW 2007, Australia; 3 College of Information System and Management, National University of Defense Technology, Changsha, Hunan, 410073 China; Beihang University, CHINA

## Abstract

Dictionary learning is a method of acquiring a collection of atoms for subsequent signal representation. Due to its excellent representation ability, dictionary learning has been widely applied in multimedia and computer vision. However, conventional dictionary learning algorithms fail to deal with multi-modal datasets. In this paper, we propose an online multi-modal robust non-negative dictionary learning (OMRNDL) algorithm to overcome this deficiency. Notably, OMRNDL casts visual tracking as a dictionary learning problem under the particle filter framework and captures the intrinsic knowledge about the target from multiple visual modalities, e.g., pixel intensity and texture information. To this end, OMRNDL adaptively learns an individual dictionary, i.e., template, for each modality from available frames, and then represents new particles over all the learned dictionaries by minimizing the fitting loss of data based on M-estimation. The resultant representation coefficient can be viewed as the common semantic representation of particles across multiple modalities, and can be utilized to track the target. OMRNDL incrementally learns the dictionary and the coefficient of each particle by using multiplicative update rules to respectively guarantee their non-negativity constraints. Experimental results on a popular challenging video benchmark validate the effectiveness of OMRNDL for visual tracking in both quantity and quality.

## Introduction

Visual tracking has been widely applied in many real-world tasks, such as video surveillance, but it poses significant challenges for computer vision community. Serious appearance variations such as illumination changes and cluttered backgrounds are obstacles to performing effective tracking in complex scenarios including multiple similar targets [1]. Various tracking techniques have been proposed to tackle these challenges, and recently, a strand of works that applies dictionary learning to visual tracking has achieved great success. Mei and Ling [2] originally proposed the *L*_1_ tracker (L1T) for robustly tracking the target under the particle filter framework. However, L1T and its variants [3, 4] suffer from one of the following drawbacks: 1) they leave the dictionary unchanged and thus often drift away from the target, or 2) traditional dictionary update strategies result in poor performance. Hence, it is essential to adaptively learn the dictionary to overcome the above drawbacks.

Dictionary learning aims to find an over-complete dictionary from training examples and learns sparse representations for these samples by using as few atoms as possible. The learned dictionary therefore significantly influences the quality of sparse representation. Recently, many dictionary learning methods have been proposed that incorporate additional constraints over either the dictionary or the sparse representations. Due to its effectiveness, dictionary learning has been widely used in computer vision such as image de-noising [5, 6], image segment [7] and image classification [8–10]. However, since the existing methods need to maintain a large collection of training samples in memory, they cannot deal with large-scale or streaming datasets such as video sequences.

Online learning has become a good alternative to improve the scalability of dictionary learning [11–15]. Marial *et al*. [11] proposed online dictionary learning based on stochastic optimization which elegantly scales well for large-scale datasets. Xie *et al*. [12] proposed projecting each descriptor into its local-coordinate system by utilizing locality constraints, followed by incrementally updating the dictionary in a gradient descent fashion. However, these methods assume that noise obeys the Gaussian distribution, and this assumption may be violated by data that is corrupted by outliers. To avoid this drawback, Lu *et al*. [13] proposed the online robust dictionary learning (ORDL) method which employs the *L*_1_ loss in data fitting. This scheme has been found to be useful for reconstructing partially occluded objects. Although these online algorithms reconstruct the objects well, they underperform in classification tasks. Recently, Yang *et al*. [14] proposed the online discriminative dictionary learning (ODDL) method for visual tracking which filters the positive particle by simultaneously minimizing a reconstruction error and a classification error. Wang *et al*. [15] proposed the online robust non-negative dictionary learning (ONNDL) method which creates a robust non-negative dictionary to adaptively model the appearance template for visual tracking in an online fashion. However, the aforementioned methods cannot deal with multi-modal datasets.

To overcome this deficiency, this paper proposes an online multi-modal robust non-negative dictionary learning (OMRNDL) method which imposes the non-negative constraint over both the dictionary and sparse coding. These non-negative constraints not only induce more sparse representation but also make the *L*_1_ regularization term differentiable. To incorporate multi-modal features, OMRNDL learns an individual non-negative dictionary over each modality of the data, and captures the intrinsic aspect of each modality of the target by sharing identical representation between these modalities. To reduce the influence of outliers, OMRNDL fits all modalities by utilizing M-estimation. OMRNDL can be easily integrated into the particle filter framework for visual tracking where each new particle can be represented by the learned sparse representation across multi-modality features. Interestingly, OMRNDL can be viewed as a multi-modal non-negative dictionary learning framework and can include ONNDL as a special case. To optimize OMRNDL, we have developed an algorithm that incrementally learns the multi-modal dictionaries and the representation coefficients by utilizing multiplicative update rules (MUR) which guarantee non-negativity constraints. The experimental results of visual tracking on twenty-two video sequences from the popular challenging video benchmark [16] suggest the effectiveness of OMRNDL in both quantity and quality.

## Analysis

There is a rich literature on visual tracking, and more details about the existing trackers can be found in the 2006 survey [17] and recent benchmark [16] comparing the state-of-the-art trackers. We briefly review the work related to our method including sparse representation-based trackers, multi-modal learning and non-negative matrix factorization.

Sparse representation has been extensively applied in visual tracking. Mei and Ling [2] proposed the *L*_1_ tracker (L1T) which is the first work to apply sparse coding to visual tracking and simply uses holistic object samples to compile the dictionary. Such templates are often vulnerable to noise because they neither take the background knowledge into account nor exploit well-studied dictionary update strategies. To incorporate the background information, Liu *et al*. [18] utilized the *K*-selection method to construct a dictionary prior to tracking. However, the dictionary remains unchanged during the tracking procedure, thus the dictionary is not adaptive to new samples. To overcome this deficiency, Jia *et al*. [19] proposed an adaptive structural local sparse appearance model to update the dictionary by detecting appearance changes and replacing the old template with the new object sample. Similarly, Zhang *et al*. [3] adopted the structure constraints in the multi-task learning framework to reject the occluded samples. In contrast, Yang *et al*. [14] presented a discriminative dictionary learning based tracking method which models the object appearance by incorporating the discriminative and reconstructive power of the dictionary. Wang *et al*. [15] proposed a robust non-negative dictionary learning method to adaptively model the appearance template in an online fashion. This tracker also utilizes the background to generate discriminative sparse coding; however, these trackers merely harness a single modality feature in dictionary learning.

Besides the aforementioned trackers, other visual tracking approaches related to our proposed method include multi-modal learning and (robust) nonnegative matrix factorization (NMF). Multi-modal learning can derive common semantic representation across multi-modal features in various fields [20–22]. It has been found that combining multi-modal features is highly beneficial for vision tasks such as facial expression generation [23], pose estimation [24], image retrieval [25], classification [26] and clustering [27, 28]. As for NMF [29, 30], it is a popular dimension reduction method. Different from traditional learning methods [31–33], it incorporates non-negative constraints over both the basis and coefficient to derive parts-based representation, which is consistent with psychological intuition to facilitate human interpretation [34]. NMF variants [35–42] and online versions [11, 43, 44] have been widely applied to computer vision to benefit from this property.

## Results

### Online Multi-modal Robust Non-negative Dictionary Learning (OMRNDL)

Due to the efficacy of combining multi-modal features, we integrate the multi-modal features into dictionary learning and propose an online multi-modal robust non-negative dictionary learning (OMRNDL) method. The tracking procedures for visual tracking-based sparse representation can be categorized as the template update and particle representation. The former depends on the dictionary learning approach, while the latter calculates the sparse coding of each particle over the learned dictionary. Both procedures can be formulated in the same way, so for brevity, OMRNDL focuses on the first procedure.

#### The Proposed Model

Assume that *n* samples are captured from the video frames. Each sample has multi-modal features ${\left\{X^{i}\in R^{m_{i}}\right\}}_{i=1}^{g}$ where *g* represents the number of modalities, and *x* ^*i*^ represents the *i*-th modal feature a *m*_*i*_-dimensional vector. We can concatenate the *i*-th modal feature of all samples into a matrix $X^{i}\in R^{m_{i}}$. Since different modalities of the same sample can be regarded as different views generated from a common basic feature, it is reasonable to assume that multiple modalities share common representation in the dictionary learning framework. In this sense, OMRNDL learns the common semantic representation *V* ∈ *R* ^*r*×*n*^ across multi-modal features and simultaneously derives multiple dictionaries *D* ^*i*^ ∈ *R* ^*m*_*i*_×*r*^ over each modality such that

$$\begin{array}{c} \min_{D^{i}\in \Omega^{+},V\geq 0}\frac{1}{2}\sum_{i=1}^{g}\alpha_{i}{\left\|X^{i}-D^{i}V\right\|}_{F}^{2}+\lambda {\left\|V\right\|}_{1}, \end{array}$$ (1)

where *α*_*i*_ trades off the *i*-th modal reconstructive error, and λ is the regularized parameter for sparse coding and Ω^+^ = {*y*|*y* ^*T*^ *y* ≤ 1, *y* ≥ 0}. According to (Eq 1), each learned dictionary can capture the distinctive aspect of each modality while the common semantic representation *V* denotes the coefficients of the examples.

The problem (Eq 1) is usually solved by using thresholding-based methods [45], but such methods cannot be extended in online fashion. We therefore impose a non-negativity constraint over the representation *V* to make the objective function in (Eq 1) differentiable as ‖*V*‖_1_ = ∑_*ij*_ *V*_*ij*_ if *V* is non-negative. We also impose non-negativity constraints over all dictionaries because the data are usually non-negative. In contrast to NMF, which learns a lower-rank basis matrix, the OMRNDL model (Eq 1) learns over-complete dictionaries to store sufficient templates for tracking.

Nevertheless, OMRNDL has some limitations: 1) it is assumed that the data noise distribution obeys Gaussian distribution in practice, and 2) it requires the entire dataset to reside in memory during the training procedure and thus is prohibitive for large-scale problems. To overcome the first deficiency, we introduce robust M-estimator functions to improve its robustness to outliers, e.g.,

$$\begin{array}{c} \min_{D^{i}\in \Omega^{+},V\geq 0}\frac{1}{2}\sum_{i=1}^{g}\alpha_{i}\sum_{j,k}^{}\varphi_{i}(x_{jk}^{i}-{\left(D^{i}V\right)}_{jk})+\lambda {\left\|V\right\|}_{1}, \end{array}$$ (2)

where *φ*_*i*_ denotes the robust M-estimator function of the *i*-th modality, and $x_{jk}^{i}$ denotes the *k*-th entry of the *j*-th example of the *i*-th modality. The robust M-estimator functions [46] such as the Huber loss function and *L*_1_ loss function have been extensively applied in various applications. We provide a multi-modal framework for robust non-negative dictionary learning which includes ONNDL as a special case. Like ONNDL, our model utilizes the Huber loss function as the robust M-estimator function, i.e.,

$$\begin{array}{c} \varphi_{i}\left(r\right)=\{\begin{array}{cc} \frac{1}{2}r^{2} & |r|<\mu \\ \mu |r|-\frac{1}{2}\mu^{2} & otherwise \end{array}, \end{array}$$ (3)

where *μ* is the parameter in the Huber loss.

The objective (Eq 2) cannot process large-scale datasets because it requires the entire set of training set to reside in the memory during the learning procedure. Thus, it cannot be applied to practical visual tracking tasks.

#### Optimization Algorithm

For efficient learning, the dictionary is updated in an online fashion and sparse coding is then calculated. Let ${\left(X^{i}\right)}^{l}\in R_{+}^{m_{i}\times n^{l}}$ denote the object samples of the *i*-th modality received at the *l*-th frame with *l* ≥ 0, where *n* ^*l*^ denotes the number of received samples, and ${\left(D^{i}\right)}^{l}\in R_{+}^{m_{i}\times r}$ denotes the dictionary of the *i*-th modality. The training set is initialized by the ground truth of the first frame. At the (*l*+1)-th frame, OMRNDL receives ${\left({\tilde{X}}^{i}\right)}^{l+1}\in R_{+}^{m_{i}\times d}$, and learns the dictionary (*D* ^*i*^)^*l*+1^ and the sparse coding *V* ^*l*+1^ on the matrix ${\left(X^{i}\right)}^{l+1}=[{\left(X^{i}\right)}^{l},{\left({\tilde{X}}^{i}\right)}^{l+1}]\in R_{+}^{m_{i}\times n^{l+1}}$, where *n* ^*l*+1^ = *n* ^*l*^ + *d* and (*X* ^*i*^)^*l*+1^ maintains samples of both the *l*-th frame and the (*l* + 1)-th frame. Like (Eq 2), we have

$$\underset{\begin{array}{l} {\left(D^{i}\right)}^{l+1}\in \Omega^{+},\\ V^{l+1}\geq 0 \end{array}}{\text{min}}\begin{array}{c} f=\frac{1}{2}\sum_{i=1}^{g}\alpha_{i}\sum_{j,k}^{}\varphi_{i}({\left(x_{jk}^{i}\right)}^{l+1}-{\left({\left(D^{i}\right)}^{l+1}V^{l+1}\right)}_{jk})+\lambda \|V^{l+1}{\|}_{1}. \end{array}$$ (4)

The optimization of (Eq 4) can employ the iterative reweighted least square (IRLS) method [47]. To optimize (Eq 4), IRLS needs to recursively iterate the following two procedures until convergence, i.e.,

$$\underset{\begin{array}{l} {\left(D^{i}\right)}^{l+1}\in \Omega^{+},\\ V^{l+1}\geq 0 \end{array}}{\text{min}}\begin{array}{c} h=\frac{1}{2}\sum_{i=1}^{g}\alpha_{i}\sum_{jk}^{}w_{j,k}^{i}{\left({\left(x_{j,k}^{i}\right)}^{l+1}-{\left({\left(D^{i}\right)}^{l+1}V^{l+1}\right)}_{jk}\right)}^{2}+\lambda \|V^{l+1}{\|}_{1}, \end{array}$$ (5)

and

$$\begin{array}{c} w_{jk}^{i}=\theta_{i}\left({\left(x_{j,k}^{i}\right)}^{l+1}-{\left({\left(D^{i}\right)}^{l+1}V^{l+1}\right)}_{jk}\right), \end{array}$$ (6)

where $w_{jk}^{i}$ is the weight of the *k*-th entry of the *j*-th sample of the *i*-th modality in the matrix form *W* ^*i*^ and the weight function *θ*_*i*_(*r*_*jk*_) of (Eq 3) is defined as follows:

$$\begin{array}{c} \theta_{i}\left(r_{jk}\right)=\{\begin{array}{cc} 1 & |r_{jk}|<\mu \\ \frac{\mu }{|r_{jk}|} & otherwise \end{array}. \end{array}$$ (7)

It is relatively easier to optimize (Eq 5) than to optimize (Eq 4). However, the objective (Eq 5) is jointly non-convex with respect to *D* ^*i*^ and *V*, where *i* = 1, …, *g*. To efficiently optimize (Eq 5), we can iteratively optimize one factor with the other factors fixed.

To distinguish the template update and the particle representation, we first optimize the dictionaries *D* ^*i*^, *i* = 1, ⋯, *g* with *V* fixed. Like [15], we update each row of (*D* ^*i*^)^*l*+1^ rather than all the rows, as for (*D* ^*i*^)^*l*+1^. We first find its derivative as follows:

$$\begin{array}{c} \frac{\partial h}{\partial {\left(D_{k●}^{i}\right)}^{l+1}}=-{\left(X^{l+1}\right)}_{k●}\Lambda_{k}^{i}{\left(V^{l+1}\right)}^{T}+{\left(D_{k●}^{i}\right)}^{l+1}V^{l+1}\Lambda_{k}^{i}{\left(V^{l+1}\right)}^{T}, \end{array}$$ (8)

where $\Lambda_{k}^{i}$ is the diagonal matrix with the diagonal elements being the *k*-th row of *W* ^*i*^.

To keep the learned historical knowledge, we utilize the projected gradient descent method to update ${\left(D_{k●}^{i}\right)}^{l+1}$:

$$\begin{array}{c} {\left(D_{k●}^{i}\right)}^{l+1}=P_{\Omega^{+}}({\left(D_{k●}^{i}\right)}^{l}-\beta \frac{\partial h}{\partial {\left(D_{k●}^{i}\right)}^{l+1}}), \end{array}$$ (9)

where *P*_Ω^+^_(*Y*) projects the matrix *Y* on the domain Ω^+^, and *β* > 0 is the step size using 0.02 in our experiments. To update the dictionary in an online fashion, we introduce the forgetting factor *ρ* > 0, and define the following auxiliary variables: ${\left(A_{k}^{i}\right)}^{l}={\left(X^{l}\right)}_{k●}\Lambda_{k}^{i}{\left(V^{l}\right)}^{T}$ and ${\left(B_{k}^{i}\right)}^{l}=V^{l}\Lambda_{k}^{i}{\left(V^{l}\right)}^{T}$, and update

$$\begin{array}{c} {\left(A_{k}^{i}\right)}^{l+1}=\rho {\left(A_{k}^{i}\right)}^{l}+{\left({\tilde{X}}^{l+1}\right)}_{k●}{\left(\Lambda_{k}^{i}\right)}^{l+1}{\left({\tilde{V}}^{l+1}\right)}^{T}, \end{array}$$ (10)

and

$$\begin{array}{c} {\left(B_{k}^{i}\right)}^{l+1}=\rho {\left(B_{k}^{i}\right)}^{l}+{\tilde{V}}^{l+1}{\left(\Lambda_{k}^{i}\right)}^{l+1}{\left({\tilde{V}}^{l+1}\right)}^{T}. \end{array}$$ (11)

According to Eqs (9), (10) and (11), we obtain

$$\begin{array}{c} {\left(D_{k●}^{i}\right)}^{l+1}=P_{\Omega }^{+}({\left(D_{k●}^{i}\right)}^{l}-\beta \left({\left(D_{k●}^{i}\right)}^{l}{\left(B_{k}^{i}\right)}^{l}-{\left(A_{k}^{i}\right)}^{l}\right). \end{array}$$ (12)

Due to the symmetric property of each dictionary, we can update these dictionaries via rule (Eq 12). Meanwhile, we merely calculate the sparse coding ${\tilde{V}}^{l+1}$ of ${\left({\tilde{X}}^{i}\right)}^{l+1}$ rather than that of (*X* ^*i*^)^*l*+1^.

To optimize *V*, we recursively iterate the following update rule until convergence

$$\begin{array}{c} V_{t+1}^{l+1}\leftarrow V_{t}^{l+1}⊗\frac{\sum_{i=1}^{g}\alpha_{i}{\left(D^{i}\right)}^{T}\left(W_{t}^{i}⊗{\left(X^{i}\right)}^{l+1}\right)}{\sum_{i=1}^{g}\alpha_{i}{\left(D^{i}\right)}^{T}\left(W_{t}^{i}⊗\left(D^{i}V_{t}^{l+1}\right)\right)}, \end{array}$$ (13)

and

$$\begin{array}{c} {\left(w_{jk}^{i}\right)}_{t+1}=\theta_{i}\left({\left(x_{j,k}^{i}\right)}^{l+1}-{\left({\left(D^{i}\right)}^{l+1}V_{t+1}^{l+1}\right)}_{jk}\right), \end{array}$$ (14)

where *t* denotes the step of the iteration round, ⊗ signifies the element-wise product, and the weight $W_{t+1}^{i}={\left(w_{jk}^{i}\right)}_{t+1}$. We summarize the multi-modal non-negative sparse coding and dictionary learning in **Table 1** and **Table 2**, respectively.

The main memory cost of **Table 2** lies in Eqs (10) and (11), thus the space complexity is $O(gr^{2}+\sum_{i=1}^{g}m^{i}r)$. Since its memory space is irrelevant to the number of samples, OMRNDL can be applied to large-scale datasets such as video sequences.

#### OMRNDL Tracker

We apply OMRNDL for visual tracking-based on the particle filter framework [48]. The particle filter framework samples a number of particles from each frame of the video according to six affine parameters: 1) horizontal translation, 2) vertical translation, 3) scale, 4) aspect ratio, 5) rotation, and 6) skewness. These are modeled by six independent zero-mean Gaussian distributions with six predefined variance values. Each particle is cropped into a fixed-size pixel array according to the shape of the object and then reshaped into a long vector. This framework tracks the target by filtering the most likely particle from each frame according to the tracking model.

**Table 1:** Multi-modal Non-negative Sparse Coding.

**Input** Multi-modal examples *X* ^*i*^ and the learned dictionary *D* ^*i*^, where *i* = 1, ⋯, *g*.
**Output** *V* and *W* ^*i*^.
1: Initialize *t* = 1, $W_{i}^{t}$ using a matrix full of one and *V*_1_.
2: **repeat**
3: Update *V*_*t*_ via (Eq 13).
4: Calculate $W_{t}^{i}$ via (Eq 14) for *i* = 1, ⋯, *g*.
5: *t* ← *t* + 1.
6: **until** {The stopping criterion $\frac{{\left\|h_{t}-h_{t-1}\right\|}_{2}}{{\left\|h_{t-1}\right\|}_{2}}<ɛ$ is satisfied, where the tolerance *ɛ* is set to 10^−3^.}
7: *V* = *V*_*t*_ and $W^{i}=W_{t}^{i}$.

**Table 2:** Online Multi-modal Robust Non-negative Dictionary Learning (OMRNDL).

**Input:** The arriving multi-modal examples ${\left({\tilde{X}}^{i}\right)}^{l+1}$, the auxiliary variables ${\left(A_{k}^{i}\right)}^{l}$ and ${\left(B_{k}^{i}\right)}^{l}$, and the learned dictionary (*D* ^*i*^)^*l*^, where *i* = 1, ⋯, *g*.
**Output:** The learned dictionaries (*D* ^*i*^)^*l*+1^, ${\left(A_{k}^{i}\right)}^{l+1}$ and ${\left(B_{k}^{i}\right)}^{l+1}$, where *i* = 1, ⋯, *g*.
1: Initialize *t* = 1, ${\left(A_{k}^{i}\right)}_{1}^{l}$ and ${\left(B_{k}^{i}\right)}_{1}^{l}$.
2: **repeat**
3: Calculate the sparse coding ${\left(\tilde{V}\right)}^{l+1}$ and the weight *W* ^*i*^ by **Table 1**.
4: Calculate ${\left(A_{k}^{i}\right)}_{t}^{l}$ and ${\left(B_{k}^{i}\right)}_{t}^{l}$ with Eqs (10) and (11), respectively.
5: Update ${\left(D^{i}\right)}_{t}^{l}$ via (Eq 12), for *i* = 1, ⋯, *g*.
6: *t* ← *t* + 1.
7: **until** {The stopping criterion $\frac{{\left\|h_{t}-h_{t-1}\right\|}_{2}}{{\left\|h_{t-1}\right\|}_{2}}<ɛ$ is satisfied, where the tolerance *ɛ* is set to 10^−2^.}
8: ${\left(D^{i}\right)}^{l+1}={\left(D^{i}\right)}_{t}^{l}$, ${\left(A^{i}\right)}^{l+1}={\left(A^{i}\right)}_{t}^{l}$ and ${\left(B^{i}\right)}^{l+1}={\left(B^{i}\right)}_{t}^{l}$.

We can choose different features as multi-modal features, such as pixel intensity, RGB color, LBP [49], SIFT [50], HoG [51], GIST [52] and SURF [53]. Generally, LBP [49] represents the texture of an image which is suitable for a tracked object on a uniform background. HoG [51] achieves success in pedestrian detection because it describes the typical profile of the person. SIFT [50] extracts the scale- and rotation-invariant features of the object which is helpful for tracking objects which have drastic changes in scale and in-plane rotation. Unlike SIFT, GIST [52] holistically represents the scale-invariant features of the object. SURF [53] is able to learn robust features quickly. To implement our OMRNDL tracker, we select image gray pixels and the corresponding textures as two modalities, i.e., *g* = 2, because they are simple and easy to implement and work with.

Like most visual trackers, our tracker assumes that the ground-truth bounding box in the first frame is available and regards it as an initial positive particle. We group the sampled particles into two categories: the positive particle and the negative particle. The positive particle contains target candidates that are consecutively filtered from each frame using the particle filter framework. The negative particles contain cluttered backgrounds that are randomly selected from all particles except the positive particle. To filter the positive particle from the total number of particles, the OMRNDL tracker learns object templates $D_{o}^{i}$ using OMRNDL (**Table 2**) on the positive particles. The OMRNDL tracker constructs background templates $D_{b}^{i}$ using the negative particles to avoid the drift problem seen in [15]. For each view, both object and background templates are adaptively updated every five frames.

By concatenating $D_{o}^{i}$ and $D_{b}^{i}$ to form a new dictionary *D* ^*i*^, the OMRNDL tracker represents a particular particle $\vec{v}$ over all the views by the linear combination of the dictionary:

$$\begin{array}{c} \min_{\vec{h}}\sum_{i=1}^{g}\alpha_{i}\sum_{jk}^{}\varphi_{i}({\vec{v}}_{jk}-{\left(D^{i}\vec{h}\right)}_{jk})+\lambda {\|\vec{h}\|}_{1}, \end{array}$$ (15)

where $D^{i}=[D_{o}^{i},D_{b}^{i}]$ and $\vec{h}$ are decomposed into two components, $\vec{h}=[{\vec{h}}_{o};{\vec{h}}_{b}]$. The objective (Eq 15) can be solved by **Table 1**. Additionally, (Eq 15) implies that the non-negative particle $\vec{v}$ can be viewed as the summation of two non-negative components, i.e., $D_{o}^{i}{\vec{h}}_{o}$ and $D_{b}^{i}{\vec{h}}_{b}$, and that these reflect the contributions of the object and background template, respectively. The more difference there is between the two components, the more likely it is that the candidate particle is positive. Therefore, the OMRNDL tracker calculates a weight for each particle over all the modalities:

$$\begin{array}{c} \rho =e^{-\delta \left(\sum_{i=1}^{g}\alpha_{i}\left(\|D_{o}^{i}{\vec{h}}_{o}{\|}_{1}-\|D_{b}^{i}{\vec{h}}_{b}{\|}_{1}\right)\right)}, \end{array}$$ (16)

where *δ* denotes a predefined constant that favors object templates rather than background templates and *e* denotes the exponential function. The higher the weight, the more likely it it that the particle contains the target, thus we select the candidate with the highest weighted particle as the tracking result. The OMRNDL tracker is presented in **Table 3**.

**Fig 1:**
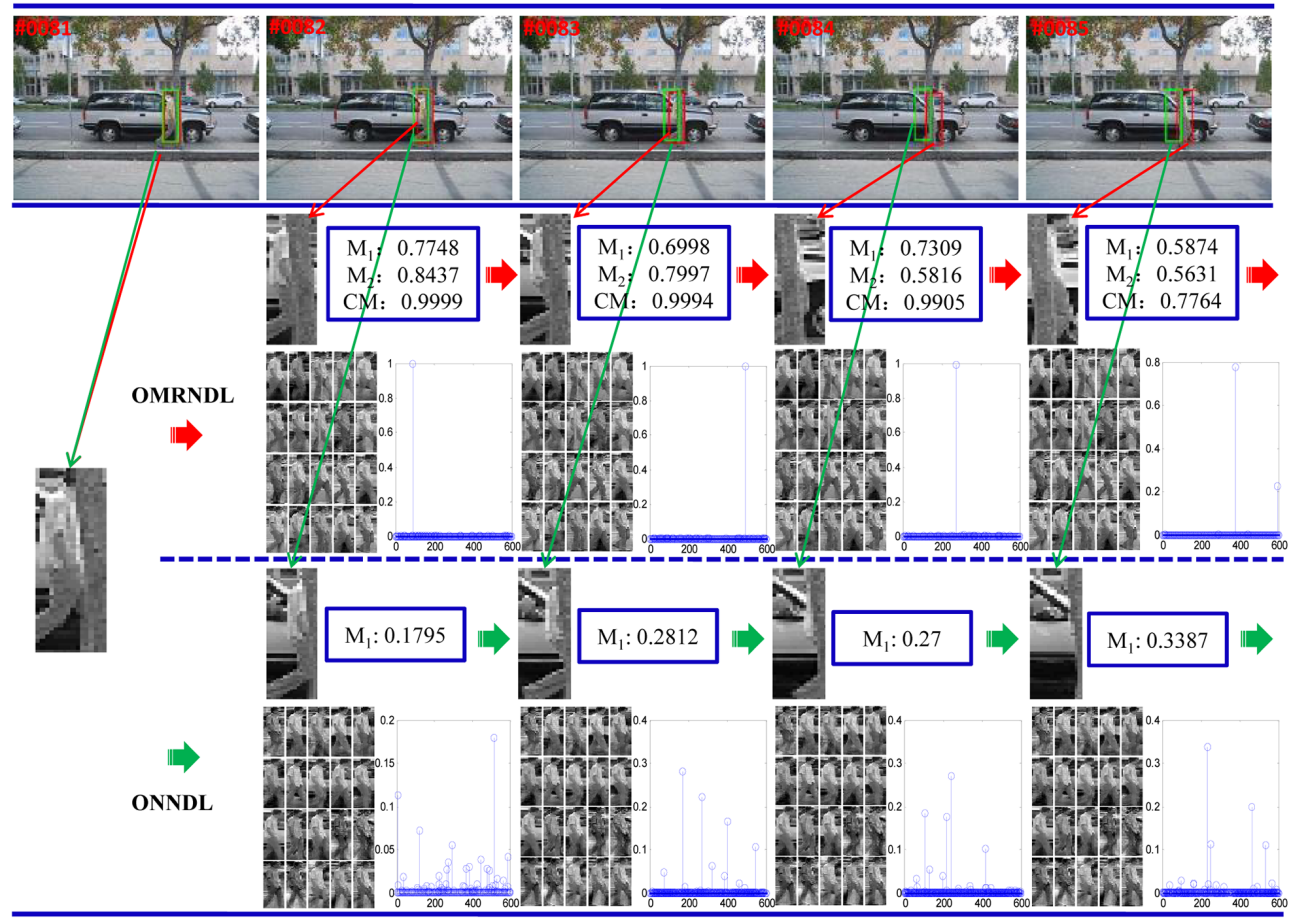
Comparisons between OMRNDL and ONNDL on the frames 81–85 of *david3*. The figure compares the weights of the most likely candidates, and the basis learned by OMRNDL and ONNDL on the frames 81–85 of david3, respectively. The first row denotes the video frames together with the bounding box obtained by OMRNDL (in red) and ONNDL (in green), respectively. The second and third rows show the tracking procedures of OMRNDL and ONNDL for determining the positive particles, respectively. The higher the weight assigned for the candidate, the more likely it is the positive particle, and thus we select the candidate with the highest weights as the tracking particle. To show the advantage of OMRNDL, each row still contains two sub-rows: 1) the selected particle and the corresponding weight, and 2) the learned basis and the weights of all the particles. *M*_1_, *M*_2_ and *CM* denote the weights of the selected particles when using the gray pixel intensity, the LBP descriptor and their combination, respectively.

To observe the importance of the integration of both modalities, we separately test OMRNDL and ONNDL to compare the weights of the particles which are crucial for the choice of the positive particles. Fig 1 depicts the tracking procedures of both OMRNDL and ONNDL over the frames 81–85 of *david3*, where the object is occluded by a tree. Due to such occlusion, ONNDL fails to select the positive particle while OMRNDL succeeds to do that by taking the advantage of combing two modalities. In Fig 1, *M*_1_, *M*_2_ and *CM* denote the weights of the particles when using the gray pixel intensities, the LBP descriptor and fuse of them, respectively.

Fig 1 shows that the *M*_1_ values of both OMRNDL and ONNDL are significantly different, and the former is much larger than the latter. This mainly results from the difference between qualities of their learned dictionaries. This also implies that OMRNDL can learn more dynamic appearances than ONNDL because of the integration of both modalities. For the selection of positive particles, the second row of Fig 1 shows that *M*_1_ in frames 82 and 83 are relatively larger but *M*_2_ are smaller, while the opposite situations happen in frames 84 and 85, i.e., either *M*_1_ or *M*_2_ is insufficient for assigning high weight for targeted particle. However, the OMRNDL tracker can consistently adopt the combined weights to assign the highest *CM* weights for the positive particles. This is because the resultant *CM* weights can avoid biasing any single modality. Thus, the OMRNDL tracker can boost the tracking performance of ONNDL by making use of multiple modalities.

**Table 3:** OMRNDL Tracker.

**Input:** The (*l* + 1)-th video frame *I*_*l*+1_.
**Output:** Tracking location *I*_*l*+1_(v*).
1: Sample a set of candidate particles ${\left\{v_{i}\right\}}_{k=1}^{K}$, where *v*_*i*_ denotes the *i*-th particle, using the particle filter framework. Then transform them into multi-modal features.
2: Update the object templates $D_{o}^{i}$ by OMRNDL according to the multi-modal features of the previously collected positive particles, if the number of particles meets the predefined constant. Otherwise, perform line 3 directly.
3: Use both the background templates $D_{b}^{i}$ and the object templates $D_{o}^{i}$ of the total modalities to yield the weights *ρ*(*I*_*l*+1_(*v*_*k*_)) of each candidate particle using (Eq 16).
4: Select the positive particle by $i=arg\underset{k=1,\cdots ,K}{\max}\rho (I_{l+1}(v_{k}))$.
5: *I*_*l*+1_(v*) = *I*_*l*+1_(*v*_*i*_).

## Experiments

This section validates the OMRNDL tracker by comparing it with IVT [54], L1T [2], TLD [55], VTD [56], Frag [57], MIL [58], NMF tracker(NMFT) [59], IOPNMF tracker(IOPNMFT) [60] and ONNDL [15] on twenty-two video sequences from the popular benchmark [16] including *basketball, bolt, boy, car4, carDark, carScale, crossing, david, david2, david3, deer, faceocc1, faceocc2, fish, football, mountainBike, shaking, skating1, trellis, walking, walking2 and woman*. These sequences are publicly available online at http://cvlab.hanyang.ac.kr/tracker_benchmark_v10.html, and include a range of appearance variations such as drastic change in illumination and the presence of occlusion. The challenges of these video sequences are listed in Table 4. It reflects that these benchmarks cover most categories of challenges. We implement the interfaces of NMFT, IOPNMFT, ONNDL and OMRNDL under the benchmark framework [16], and conduct the experiments by running the benchmark code.

**Table 4:** Challenges of Tested Sequences.

Video	Illumination	Occlusion	Scaling	Motion	Cluttering	Rotation	Deformation
*basketball*	✓	✓			✓	✓	✓
*bolt*		✓				✓	✓
*boy*			✓	✓	✓	✓	
*car4*	✓		✓	✓	✓		
*carDark*	✓				✓		
*carScale*		✓	✓	✓		✓	
*crossing*			✓	✓	✓	✓	✓
*david*	✓	✓	✓			✓	
*david2*						✓	
*david3*		✓			✓	✓	✓
*deer*				✓	✓	✓	
*faceocc1*		✓					
*faceocc2*	✓	✓				✓	
*fish*	✓						
*football*		✓			✓	✓	
*mountainBike*					✓	✓	
*shaking*	✓		✓		✓	✓	
*skating1*	✓	✓	✓		✓	✓	✓
*trellis*	✓		✓		✓	✓	
*walking*		✓	✓				✓
*walking2*		✓	✓				
*woman*	✓	✓	✓	✓		✓	

Each row stands for a video sequence while each column denotes a challenge. Thus, the location of ‘✓’ indicates that the video sequence covers the corresponding challenge.

Our tracker was implemented in Matlab R2010a on a workstation which contains four 3.4GHz Intel (R) Core (TM) processors and 8GB RAM. To make use of multi-modal features, we extracted two types of features: pixel intensities and local binary patterns (LBP, [49]). For the OMRNDL tracker, we set all parameters *α*_*i*_ from {0.5, 1, 2}, λ = 1 and *ρ* = 0.99 in our experiments. Its current implementation runs at the rate of about 5–20 frames per second (fps).

### Qualitative Comparison

**Fig 2:**
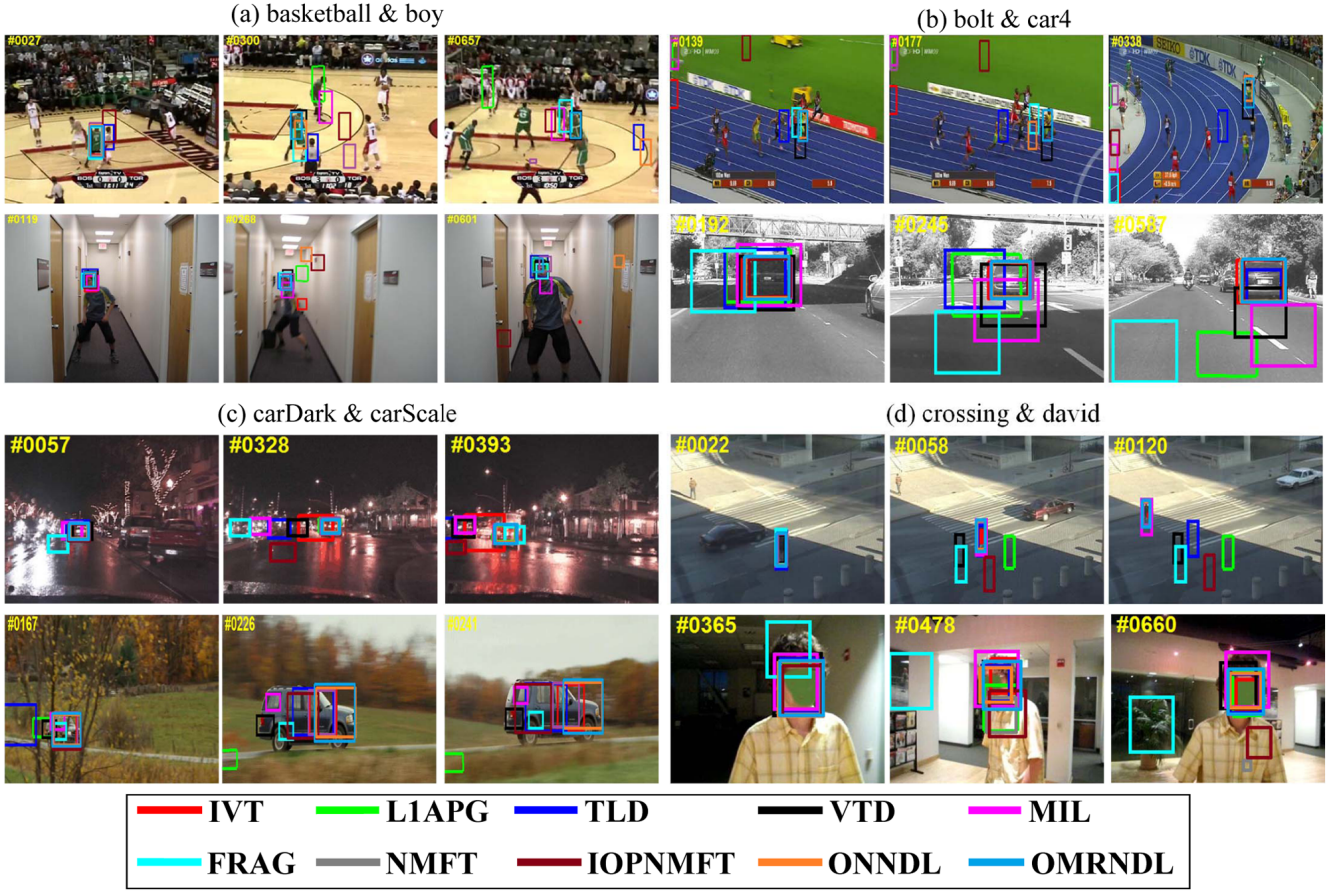
The tracking results of ten trackers in terms of the bounding box. The tracking results of IVT, L1T, TLD, VTD, Frag, MIL, NMFT, IOPNMFT, ONNDL and OMRNDL on (a) *basketball* & *boy*, (b) *bolt* & *car4*, (c) *carDark* & *carScale*, and (d) *crossing* & *david*.

**Fig 3:**
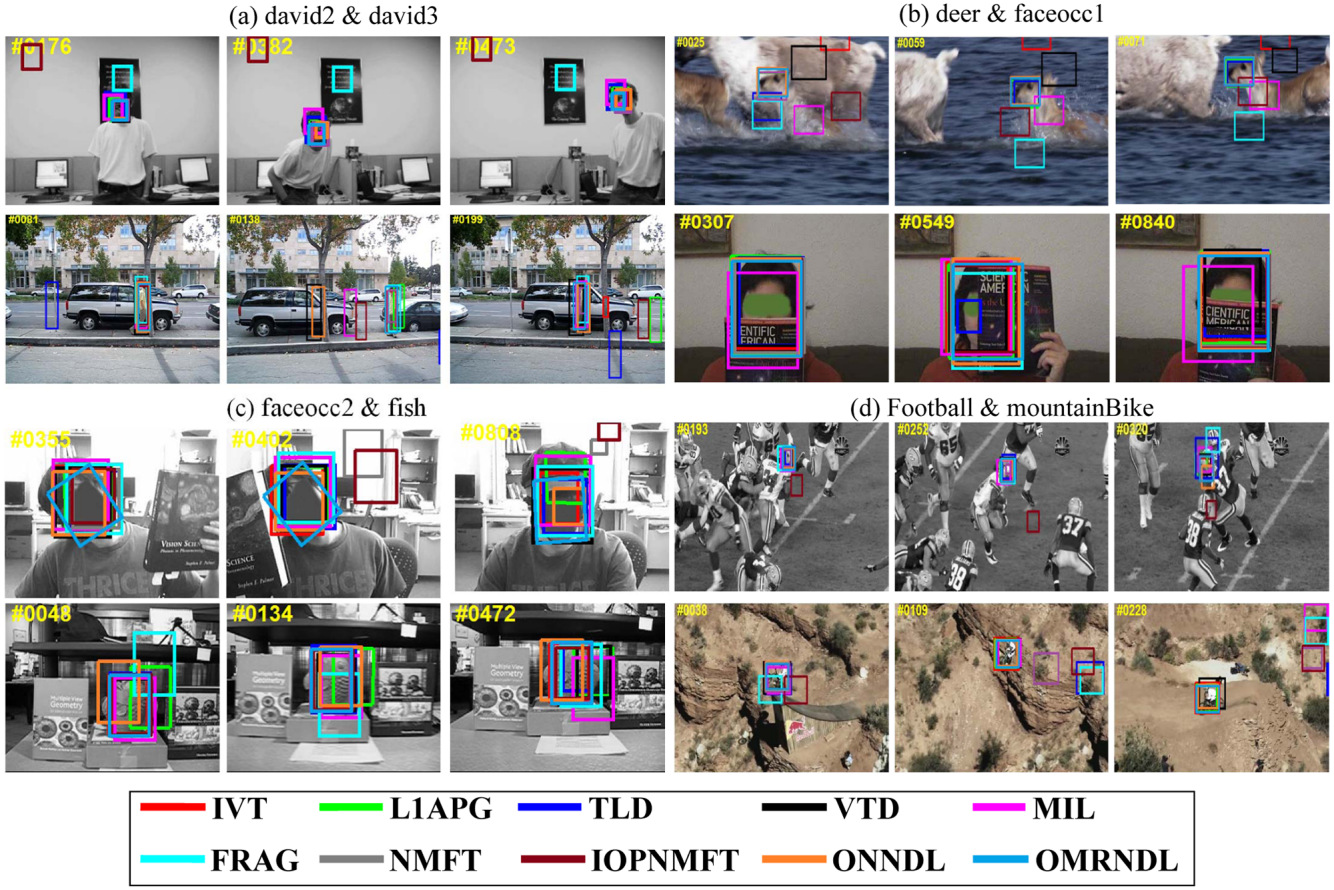
The tracking results of ten trackers in terms of the bounding box. The tracking results of IVT, L1T, TLD, VTD, Frag, MIL, NMFT, IOPNMFT, ONNDL and OMRNDL on (a) *david2* & *david3*, (b) *deer* & *faceocc1*, (c) *faceocc2* & *fish*, and (d) *football* & *mountainBike*.

**Fig 4:**
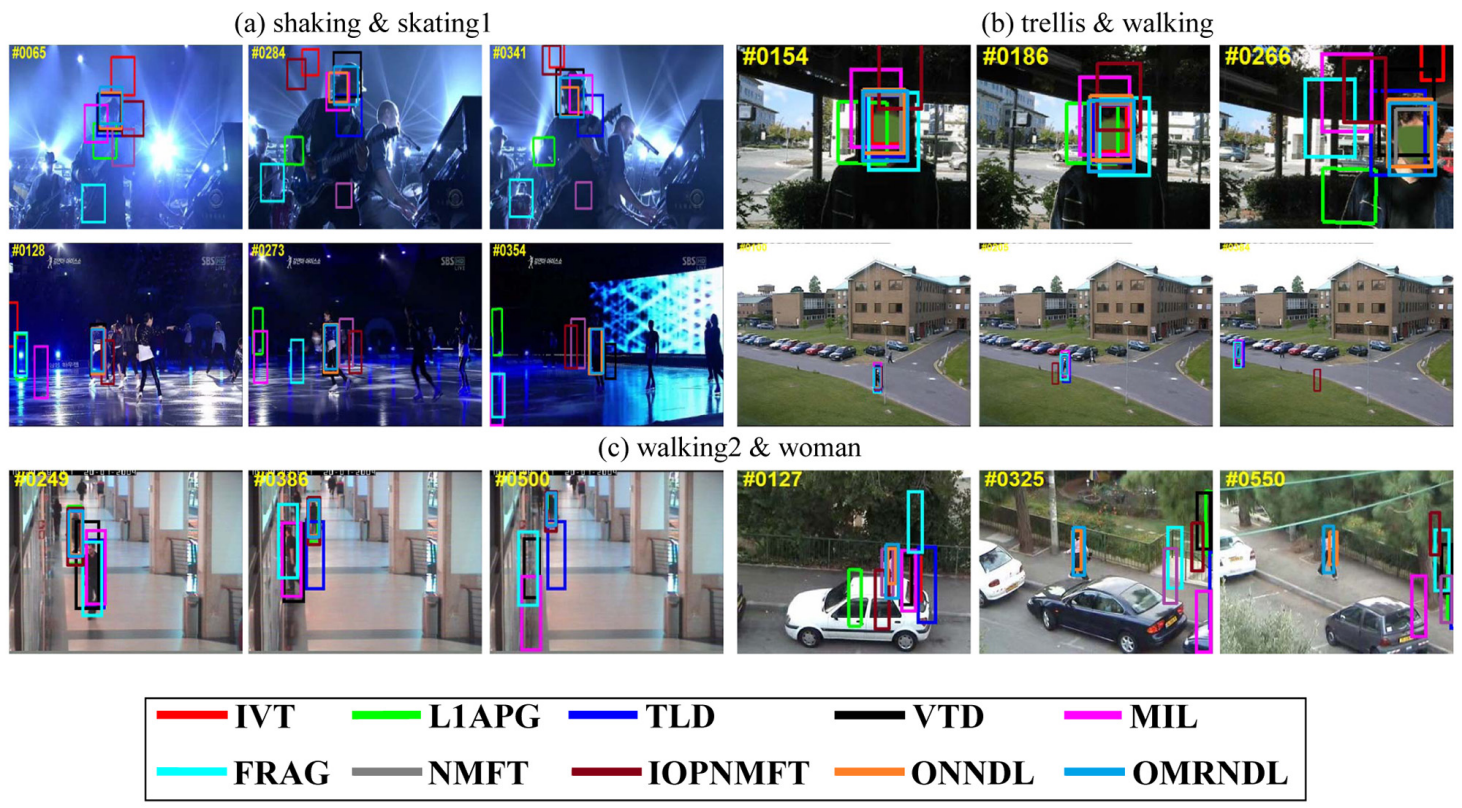
The tracking results of ten trackers in terms of the bounding box. The tracking results of IVT, L1T, TLD, VTD, Frag, MIL, NMFT, IOPNMFT, ONNDL and OMRNDL on (a) *shaking* & *skating1*, (b) *trellis* & *walking*, and (c) *walking2* & *woman*.

Fig 2 shows key frame bounding boxes reported by all ten trackers on the 22 video sequences. In the basketball, bolt and boy sequences, the tracked targets are persons moving very quickly. In *basketball*, the video sequences exhibit background clutter when many players run together. In *bolt*, the tracked object is small with low resolution and shows drastic changes in pose. In *boy*, the head of the target changes quickly. Fig 2(a) and 2(b) shows that our OMRNDL performs consistently well in all three video sequences. In the *car4*, *carDark* and *carScale* sequences, moving cars are being driven on the road in day, night and field environments. In *car4*, the video sequences undergo serious illumination changes when the vehicle runs through a tunnel or under trees. In *carDark*, the tracked car is small with low contrast and small changes in illumination. In *carScale*, the scale of the target car changes drastically. Fig 2(b) and 2(c) shows that NMFT, IOPNMFT, ONNDL and OMRNDL succeed in tracking the target in all three video sequences. In the *crossing* sequence, the target walks cross the road in dark shade, which blurs the target. Fig 2(d) shows that IVT, MIL, NMFT and OMRNDL remove the effect of the dark shade to successfully track the person. In *david*, *david2* and *david3*, the video sequences record David in indoor and outdoor environments. According to Figs 2(d) and 3(a), both ONNDL and OMRNDL benefit from adaptive dictionaries and consistently demonstrate stable performance in *david* and *david2*. In *david3*, although he undergoes the complete occlusion when David walks through the tree, OMRNDL still tracks him successfully. The deer sequences shown in the first row of Fig 3(b) track the head of a fast moving deer. The background easily induces drift in the trackers due to the similarity of several deer. OMRNDL succeeds in tracking the object completely. In both *faceocclu1* and *faceocclu2*, shown in Fig 3(b) and 3(c), the drastic occlusion changes result in extensive drift of the trackers in some frames. However, both ONNDL and OMRNDL perform stably. In *fish*, the unstable camera makes the target appear to be moving quickly. Fig 3(c) shows that OMRNDL performs stably. In *football*, the tracked hat of the football player is often cluttered by the similar background. As shown in Fig 3(d), OMRNDL, L1T and Frag perform well in this sequence compared with the other trackers. In *mountainBike*, OMRNDL still performs well. In *shaking* and *skating1*, the tracked targets of three sequences are exposed to drastic changes in illumination on the stage. Row (a) of Fig 4 shows that OMRNDL consistently performs better than other trackers. In *trellis*, the target walks in a black background while undergoing a change in illumination. The dark background causes many trackers to drift, but OMRNDL still performs well. In *walking*, a man undergoes a scale change in the scene, while *walking2* includes a walker walking down an aisle. However, the second row of Fig 4(b) shows that most trackers perform well in *walking*. The target in *walking 2* undergoes partial occlusion when someone walks behind him. In *woman*, the tracked woman is partially occluded by cars. This often induces drift in many trackers, but both ONNDL and OMRNDL succeed in tracking the subject.

### Quantitative Comparison

To quantify the performance of OMRNDL for visual tracking, we evaluate the trackers compared [2, 15, 54–58] in terms of success rate and precision [16]. The OMRNDL tracker reports high success rates for most of the tested videos under different attributions, such as variations in illumination and scale.

**Fig 5:**
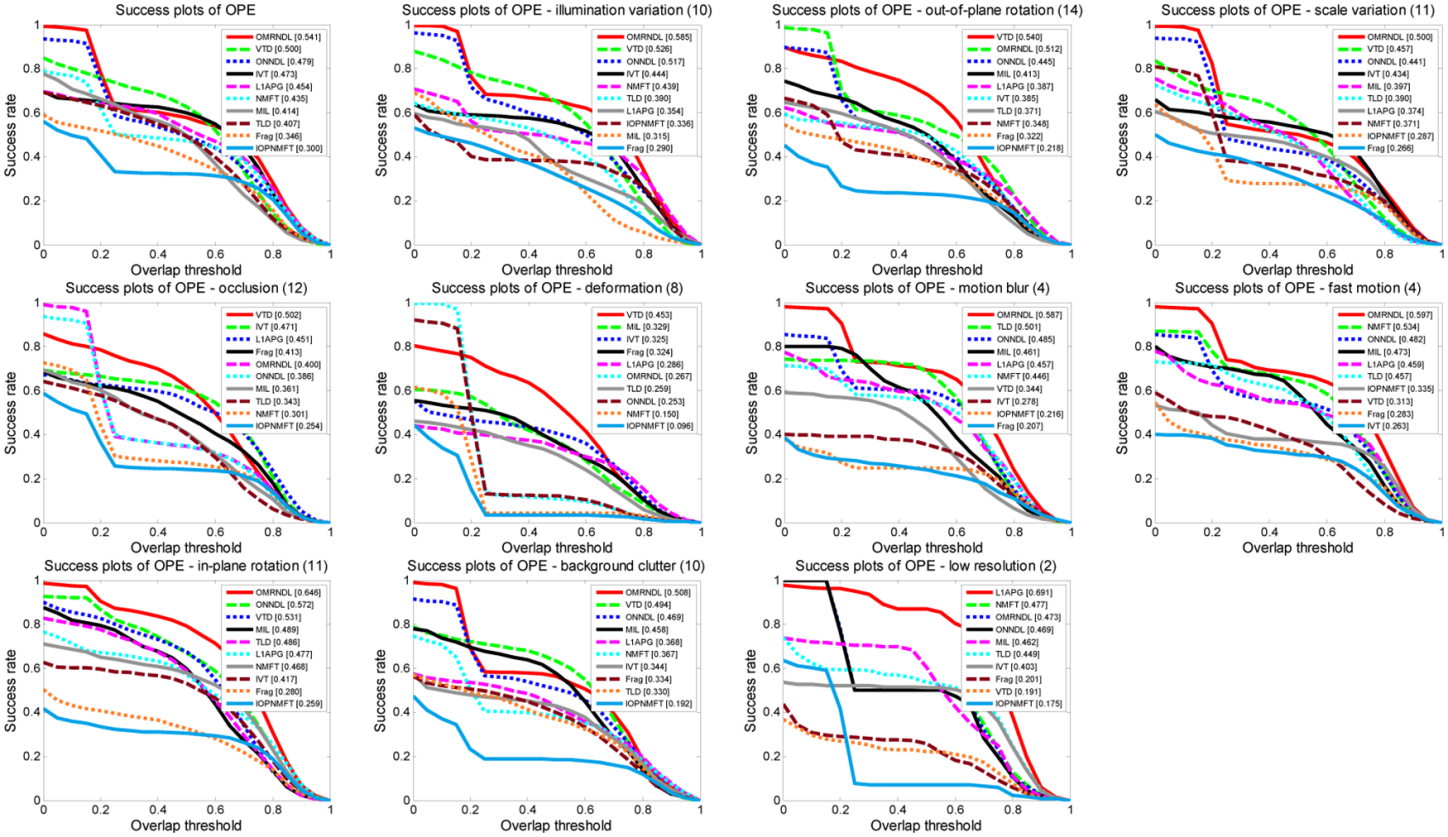
Success rate of ten trackers versus different thresholds under different attributions on twenty-two video sequences. Success rate of ten trackers versus different thresholds under different attributions including illumination variation, rotation, scale variation, occlusion, deformation, motion blur, fast motion, background clutter and low resolution on twenty-two video sequences.

Fig 5 compares the success rate of ten tested trackers on 22 video sequences. OMRNDL performs very better compared with the other trackers under most of attributions such as motion blur and low resolution. It also shows that OMRNDL can effectively handle illumination variations, scale changes, background clutter, motion blur, *etc*., and thus it can works well for object tracking. This is attributed to the integration among multi-modal features and effective representation power of the learned robust dictionaries.

**Fig 6:**
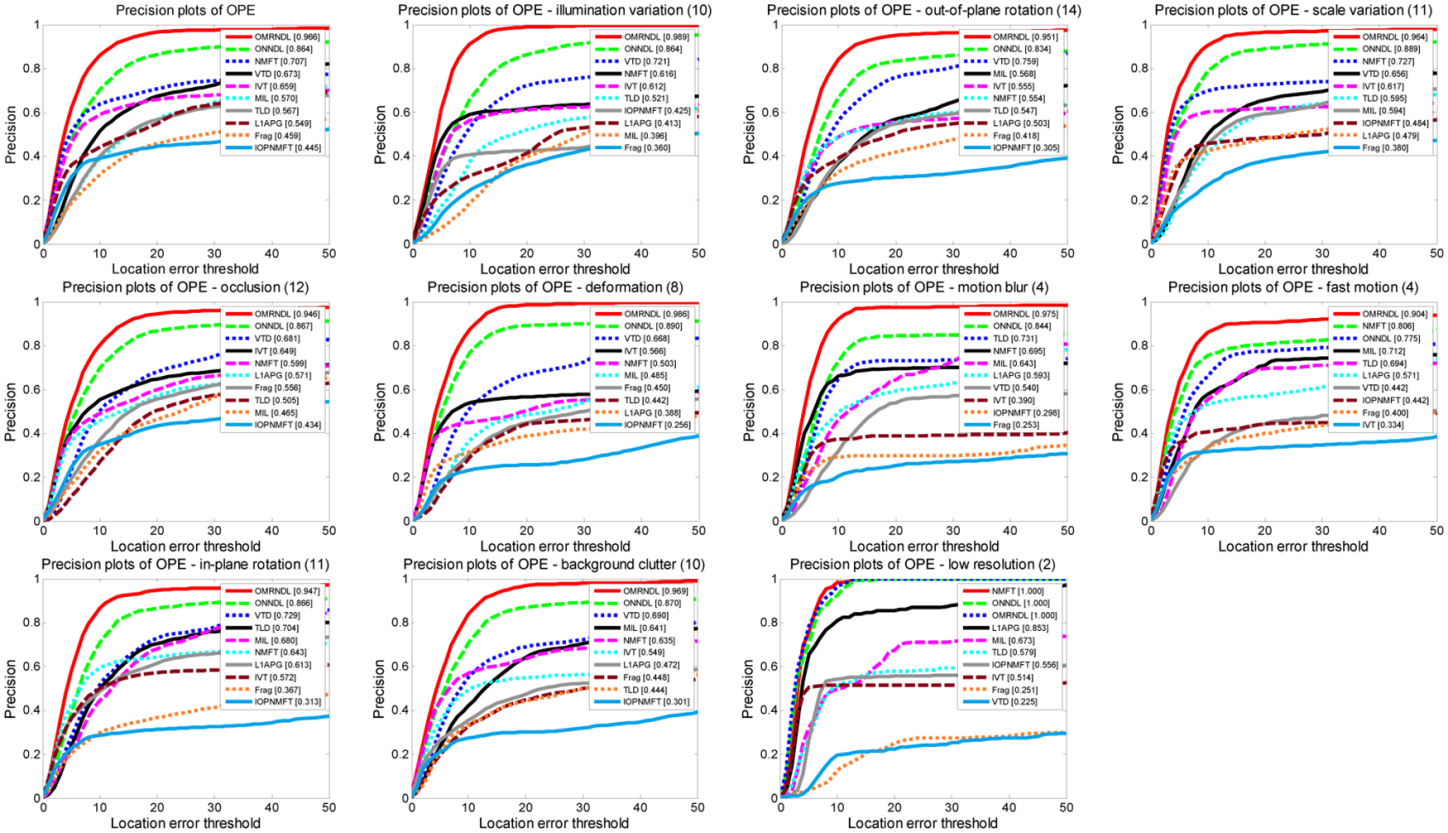
Precision of ten trackers versus different thresholds under different attributions on twenty-two video sequences. Precision of ten trackers versus different thresholds under different attributions including illumination variation, rotation, scale variation, occlusion, deformation, motion blur, fast motion, background clutter and low resolution on twenty-two video sequences.

The precision of ten tested trackers on 22 video sequences is shown in Fig 6. OMRNDL achieves consistently better performance than the other trackers under different attributions and has the highest precision. It also indicates that OMRNDL can tightly enclose the targeted objects in all the tested sequences because it can robustly learn dictionaries for each modality to represent the tracked object in an adaptive manner. This induces the robustness of OMRNDL to different challenges and further avoids the object drifting.

In summary, the OMRNDL tracker outperforms the other trackers in terms of both success rate and precision, and performs consistently well on a variety of videos.

## Conclusion

This paper proposes an efficient online multi-modal robust dictionary learning (OMRNDL) method to learn a non-negative dictionary for each view in an online fashion. OMRNDL learns the common semantic representation from multiple visual cues, and thus enhances the robustness of the sparse coding to outliers, e.g., particles that contain no target. Since OMRNDL keeps the memory overheads constant when dealing with streaming datasets, it is well-suited to tracking a single target on flying videos. Experimental results on a well-known challenging video benchmark suggest its effectiveness by both quantitative comparison and qualitative comparison.
